# Applying the workload indicators of staffing needs method in nursing health workforce planning: evidences from four hospitals in Vietnam

**DOI:** 10.1186/s12960-021-00668-y

**Published:** 2022-01-28

**Authors:** Thu Thi Hoai Nguyen, Hung Thanh Phung, Anh Thi My Bui

**Affiliations:** 1 Department of Health Management and Organization, Institute for Preventive Medicine and Public Health, Hanoi Medical University, 1A Ton That Tung Street, Dong Da District, 10000 Hanoi, Vietnam; 2 Department of Hospital Management, Health Management Training Institute, Hanoi University of Public Health, 1A Duc Thang Road, Duc Thang Ward, North Tu Liem District, 10000 Hanoi, Vietnam

**Keywords:** Workload Indicators of Staffing Need, WISN, Nursing workforce, Human resource for health, Vietnam

## Abstract

**Background:**

Vietnam has encountered difficulties in ensuring an adequate and equitable distribution of health workforce. The traditional staffing norms stated in the Circular 08/TT-BYT issued in 2007 based solely on population or institutional size and do not adequately take into consideration the variations of need such as population density, mortality and morbidity patterns. To address this problem, more rigorous approaches are needed to determine the number of personnel in health facilities. One such approach is Workload Indicators of Staffing Need (WISN) developed by the World Health Organization (WHO), a facility-based workforce planning method that assists managers in defining the responsibilities of different workforce categories and improving the appropriateness and efficiency of a staff mix.

**Methods:**

This study applied the WISN approach and was employed in 22 clinical departments at four hospitals in Vietnam between 2015 and 2018. 22 targeted group discussions involving nurses were conducted. Hospital personnel records have been retrieved. The data were analyzed according to WISN instructions.

**Results:**

Of the 22 departments, there was a shortage of 1 to 2 nurses in 10 departments, with WISN ratios ranging between 0.88 and 0.95. Only 01 clinical colleges at Can Tho Hospital lacked 05 nurses, facing a high workload with a WISN ratio of 0.78. Administrative time represented 20–40% of the total work time of a nurse. In comparison, nurses at Can Tho Hospital spent time on administration from 24 onwards. 5–41.7% of their working time while nurses at Thanh Hoa Hospital spent 21–33%.

**Conclusions:**

The application of the WISN enabled health managers to analyze the workload of nurses, calculate staffing needs, and thus effectively contribute to the workforce planning process. It is expected that the results of this research will encourage the use of the WISN tool in other hospitals and health facilities across the health system. At provincial and national levels, this study provides important evidence to help policy makers develop guidelines for personnel norms for health facilities in the context of limited resources, while the existing regulation is no longer appropriate.

**Supplementary Information:**

The online version contains supplementary material available at 10.1186/s12960-021-00668-y.

## Background

According to the health care framework of the six building-blocks of the World Health Organization (WHO) [1], human resources play an important role in providing and ensuring quality health services to the community, as well as is one of factors affecting the effectiveness and efficiency of health systems in country. Recently, the developing countries, particularly in low and middle income countries (LMICs) are facing the need for trained health‑care staffs to meet with the increasing demand for quality health services that could led to a pressure on managers in effectively allocating their human resources [2–5].

As a human resource to address health challenges, the World Health Organization developed the Staffing Needs Workload Indicators (WISN) in the late 1990s [4]. The advantages of the WISN method indicated in some studies that was the calculation basis on actual work that has made it a completely objective tool for determining the desirable level of human resources. In addition, the application of the WISN could lead to efficient use of resources and enhance the quality of services. [5, 6].

The WISN method has been used in practice in several developing countries to determine staffing requirements for multiple categories by level of care and geographic areas. In Uganda, for instance, the first WISN experiment was carried out for different categories of health staffs working in health facilities, then the second WISN application focused on nurses, and nursing assistants in a tertiary-level hospital. On the other hand, the WISN in Mozambique and Indonesia targeted maternal and child health workers, midwives, clinicians and nurses in rural and urban hospitals or health centers [7]. The WISN application has contributed to appropriate evidence-based health workforce policy development, planning and management. WISN has been useful for health care managers in the staffing decision-making process; determining the required number of specific type health staff for health services in each facility; estimating work pressure on health workers; and reallocating or managing the transfer of functions—transferring and sharing tasks; planning for future staffing of health care service delivery based on anticipated workload of health facilities [5, 8].

Vietnam has struggled to ensure an appropriate and equitable distribution of the health workforce [9]. The traditional staffing standards set out in Circular 08/TT-BYT released in 2007 [10] based solely on population or institutional size and does not adequately reflect changes in needs, such as population density, mortality and morbidity patterns. To solve this problem, it is necessary to seek more rigorous approaches to determining staffing levels in healthcare facilities. The main objective of this study is to assess and calculate the current workloads and staffing needs of nurses in 4 selected hospitals in Vietnam, using workload indicators of staffing needs. The WISN method is based on the work undertaken by health staffs (medical doctors and nurses) and is used in this context to estimate human resources availability for medical activities at the provincial hospital level.

## Methods

### Study design

The study applied the Workload Indicators of Staffing Need (WISN) manual [11]. The WISN method is a human resource tool for staffing planning and decision making that considers certain information in the calculation of staffing requirements for health care services and activities. The information requested includes the components of the workload (i.e. the common activities performed by a cadre on basic daily health services), the time of activities standards (i.e. the time it takes a cadre to perform their activities standards), the available working time (i.e. the available time in one year for a cadre to complete their work) and the annual workload statistic (i.e. the annual statistic of health services delivery in healthcare facility). Therefore, the study was used with the combination of quantitative and qualitative methods and secondary data retrieval to gather key data for the WISN calculation.

### Study sites

The study was conducted from 01/2015 to 12/2018 in 4 provincial general hospitals in Bac Giang, Thanh Hoa (in the North of Vietnam) and Can Tho, Dong Thap (in the South of Vietnam).

### Object of research and data collection

#### Phase 1

Secondary data collection: the study used data collection checklist to collect hospital reports on personnel, hospital statistics in 2015–2016 (Bac Giang, Thanh Hoa hospitals), in 2017–2018 (Can Tho and Dong Thap hospitals).

#### Phase 2

##### Quantitative research

The three-category-of-nurse (including clinical nurse, administrative nurse and pharmaceutical nurse) was identify in each department, 2 nurses in each category were selected in this phase. Total of 132 selected nurses in 22 clinical departments in 4 hospitals were interviewed about working hours and tasks, using a semi-structured questionnaire. The questionnaire was designed according to the WISN manual construction and focused on 3 workload components (i.e. clinical activities, administrative/ management activities and other activities (e.g. extracurricular activities, musical performance, youth and union activities in hospitals, etc.)

#### Phase 3

Qualitative research, the study conducted 22 focused group discussions (FGDs) with nurses working in 22 selected faculties to identify and validate workload components, activity standards and time standard for performing specific activities. Each FGD included 5–6 nurses (head nurses, clinical nurses and administrative nurses).

### Data collection

The study followed WHO guidelines on steps to apply the WISN toolkit [11]. To determine available working time (AWT), the study used a secondary data checklist to collect working hours for nurses in the previous year of the research year. To identify workload components, the study sent questionnaires to nurses of selected faculties, then organized FGDs and collected advices from health experts from Provincial Department of Health and stakeholders (hospital directors, head nurses, clinical nurses and administrative nurses) to identify and validate workload components (i.e. clinical activities, administration/management activities and other activities. In particular, the clinical activities focused on 13 standard routine activities of nurses in internal medicine and surgery block in hospitals.

The FGDs were also at the aim to define activity standards. At all four hospitals, the research used an observational method to determine nursing activity standards. Each activity was observed a minimum of 10 times by head nurses and the research team to accurately estimate the duration of nursing activity standards.

The study also collected reports on the results of nursing activities during the year in order to calculate standard workloads, allowance factors and and total required number of staff based on WISN.

### Data analysis

The quantitative data was checked and entered into the Excel software for analysis according to the instructions in the WISN toolkit.

#### WISN technique and calculations

According to WISN method, the required input for calculation of staffing requirements including the AWT, number of current staff, workload components, time of activity standards and annual workload of health services.

1) $${\text{AWT }} = {\text{ A}} - ({\text{B}} + {\text{C}} + {\text{D}} + {\text{E}})$$

In which: A: the number of available working days in a year; B: the number of public holidays; C = the number of day-off due to annual leave; D = the number of day-off due to sickness; E = the number of day-off due to other leaves;

2) Activity standard (AS): Time necessary for a cadre to perform their standard activity. AS to calculate the standard workload, category allowance factor (CAF) and individual allowance factor (IAF).

$${\text{Standard workload }} = {\text{Available Working Time}}/{\text{ Activity Standard}}$$

(The activity standard was obtained through key informant interview with head nurses).

3) Category allowance factor (CAF): This was calculated by summing up the percentages of time it takes all members of the staff category to perform activities for which the annual statistics were not available known as category allowance standard (CAS). The CAF was calculated by using the formula: ${\text{CAF }} = { 1}/ \, \left( {{1 } - \, \Sigma {\text{CAS}}\% } \right)$.

4) Individual allowance factor (IAF) was used to derive the individual allowance factor using the formula: ${\text{IAF }} = {\text{ IAS}}/{\text{AWT}}$.

In which, individual allowance standard (IAS) was obtained by calculating how much time additional activities of certain cadre require.

5) Workload-Based Staffing Requirements: The results obtained from the above variables were then used to compute the workload-based staffing requirements using the formula:

$${\mathbf{WISN}} \, {\mathbf{staff}} \, {\mathbf{requirement}}: \, = \, \hbox{-}\left[ {\left( {{\mathbf{Annual}} \, {\mathbf{workload}} \, {\mathbf{x}} \, {\mathbf{CAF}}} \right)/{\mathbf{Standard}} \, {\mathbf{workload}}} \right] \, + \, {\mathbf{IAF}}$$

For qualitative data, all information from FGDs was transcribed and analyzed by content analysis techniques. The content was analyzed to provide comprehensive evidence and reconfirm nurse workload components and work standards.

### Ethical consideration

The study was approved by the Institutional Review Board of the Hanoi University of Public Health (Approval number 017-173/DD-YTCC).

## Results

Table 1 described general information of 04 selected hospitals. The research collected data at Bac Giang and Thanh Hoa general hospitals in 2015–2016, at Can Tho central hospital and Dong Thap general hospital in 2017–2018. There were 22 clinical faculties studied, including 11 faculties of surgery and 11 faculties of internal medicine. In general, the available working time (AWT) of hospitals ranged from 1850 h to 2000 h per year, varied by hospital due to the difference in number of days off of faculties. In Can Tho central hospital for instance, the AWT of nuses working in Neurology faculty was 1633 h because the nurses in this faculty are required to work 7 h per day.

**Table 1:** General information of the selected hospitals

Year of research	Provincial general hospital			
	Bac Giang	Thanh Hoa	Can Tho	Dong Thap
	2015	2015	2017	2018
Number of faculty in research	7	6	5	4
Surgery	3	3	3	2
Internal medicine	4	3	2	2
Available working time (AWT) (hours)	1856–1988	1976–2008	1633–1989	1958–2021
Number of inpatients in each faculty/year	1545–5773	2012–5845	3478–7777	1633–7003
Number of nurses in each faculty	11–21	15–21	16–23	11–16
Total number of inpatient treatment days	18 923–44 739	18139–49 963	15 625–35 455	13 560–63 805

The number of patients hospitalized annually at each faculty varied from hospital to hospital. In particular, the highest number of inpatients per year was recorded at Can Tho central hospital (from 3478 to 7777 inpatients) and at Dong Thap general hospitals (from 1633 to 7003 inpatients). In comparison, the range of inpatient number per year was lower than in Thanh Hoa hospital and Bac Giang hospital from 2012 to 5845 inpatients and from 1545 to 5773, respectively. The number of nurses in each department also varied among hospitals. Can Tho central hospital has a largest number of nurses from 16 to 23 nurses, followed by Thanh Hoa hospital (15–21 nurses in each faculty) and Bac Giang hospital (11–21 nurses in each faculty). Otherwise, Dong Thap general hospital had a small number of nurses, from 11 to 16 nurses in each faculty. According to hospital statistics, the total number of inpatient treatment days in each faculty was highest in Dong Thap general hospital (from 13 560 to 63 805 days), meanwhile it reported only from 15 625 to 35 455 days for inpatient treatment in Can Tho central hospital.

Table 2 described the standards for nursing activities. The findings showed that the time allotted to the normal activity of each faculty in hospitals varied. For example, measuring survival indicators by a nurses working in Bac Giang and Thanh Hoa general hospitals was reported to take 5 min, however in Can Tho and Dong Thap general hospitals, this activity was completed in only 3 min. Similarly in terms of standard time to perform intravenous injection, in Bac Giang and Thanh Hoa general hospitals nurses need 5 min, more than those reported in Can Tho and Dong Thap general hospitals. Meanwhile, standard time of several activities in Can Tho, Dong Thap took longer than those in Bac Giang and Thanh Hoa general hospitals. For example, a standard time for blood transfusion by nurses in Bac Giang general hospital was 15 min, in Thanh Hoa and Can Tho general hospitals was 30 min; and from 30 to 40 min in Dong Thap general hospital. Even within a hospital, the standard time for performing an activity within the faculties was variable.

**Table 2:** Comparison of common standard activities of nurses for performing specialized services by hospitals

Activities	Unit	Provincial general hospital			
		Bac Giang	Thanh Hoa	Can Tho	Dong Thap
Monitoring patient with ICU levels	Mins/patient	15/30	15/20/30	15/30	15/30
Medical procedures	Mins/patient	30–100	15/30	5/30/120	10/30/60
Preparation of patients before and after surgery	Mins/patient	30	25/30	10/15/20	10,15
Measuring survival indicators	Mins/time	5	5	3	3
Taking additional blood	Mins/time	5	3	5	5
Infusion/Transfusion	Mins/time	5	5	6,7	7
Intravenous injection	Mins/time	5	5	3–4	3–4
Intramuscular injection	Mins/time	3	3	2	2
Subcutaneously injection	Mins/time	3	2	2	2
Blood transfusion	Mins/time	15	30	30	30/40
Sonde fastrique	Mins/time	5	5	5/6	8–10
Sonde Urinaire	Mins/time	7	5	5–7	8–10
Dressing	Mins/time	10	10	6–9	8

Table 3 describes the time spent on clinical services and administrative activities. The results showed that the time of administrative /management activities accounts for 20–40% of the total working time of a nurse. The faculties have different proportions of time for these activities. In comparion to other selected hospitals, nurses in Can Tho hospital spent the most time on administrative/management activities, from 24.5 to 41.7% of their working time. Nurses in Thanh Hoa spent between 21 and 33% of their working time on administrative/management activities, including daily professional meetings in faculty, meeting of hospital, patient record completion, writing reports, etc.

**Table 3:** The time spent to perform clinical services and administrative-management activities

	Proportion of time allocated for clinical services and administrative/management activities			
	Bac Giang (%)	Thanh Hoa (%)	Can Tho (%)	Dong Thap (%)
Clinical services	63.2–74.4	66.8–79.1	61.3–75.5	67.2–75.6
Administrative/ management activities, other activities	25.6–36.8	20.9–33.2	24.5–41.7	24.4–32.8

Table 4 described the staff needs of the faculties following calculation using the WISN tool. The results showed that out of 22 clinical faculties in 04 general hospitals surveyed, 10 faculties had a shortage of nurses. The shortage of nurses was mainly occurred in the Internal Medicine faculties (7 faculties including 3 faculties at Bac Giang hospital (General medical, Cardiology, Gastroenterology), 2 faculties at Thanh Hoa hospital (General medical, Respiratory), 1 faculty at Can Tho hospital (General medical) and 2 faculties at Dong Thap hospital (General medical, Respiratory) whereas there were only 2 Surgical faculties (2 General Surgery faculties at Can Tho and Dong Thap hospital) did not have enough nurses. These understaffed faculties should be completed between 01 and 02 nurses each. Workload pressures in these faculties were not significant, with WISN ratios ranging from 0.88 to 0.95.

**Table 4:** Required number of nursing staff based on WISN and WISN ratio

	Provincial general hospital			
	Bac Giang	Thanh Hoa	Can Tho	Dong Thap
Number of understaffed faculties	3	2	2	3
Internal medicine	3	2	1	2
Surgery	0	0	1	1
Shortage in understaffed faculties	1–2	0	2/5	1
WISN ratio in understaffed faculties	0.88–0.95	0.88–0.95	0.78–0.92	0.91–0.92
Number of adequate staffed faculties	1	0	1	1
Internal medicine	0	0	1	0
Surgery	1	0	0	1
Number of overstaffed faculties	3	4	2	0
Internal medicine	1	1	0	0
Surgery	2	3	2	0
Excess in overstaffed faculties	1/3	2/3	2	–

A single clinical faculty at Can Tho general hospital lacked 05 nurses, facing a high workload with a WISN ratio of 0.78. Three of the 22 faculties had enough staff, including 01 in internal medicine and 2 in surgery. The number of overstaffed faculties was 09 faculties, including 07 Surgical faculties and 2 Internal medicine faculties, with the excess number of nurses ranging from 01 to 03, depending on the faculties. Thus, we could see that if the shortage of nurses occurred mainly in the faculties of internal medicine, the excess of nurses often ended up in the faculties of surgery. Dong Thap general hospital did not have adequate number of staffs in almost clinical faculties, while at Thanh Hoa Hospital general hosptial, 2/3 of the clinical faculties (equivalent to 4 faculties) had excess human resources.

## Discussion

The aim of the study was to assess and calculate the current workloads and staffing needs of nurses in 4 selected hospitals and provide lessons learnt for application of WISN in the limited-resource context such Vietnam. There were 10 faculties among 22 faculties lacked of nurses, the mainly shortage of nurses occurred in the Internal Medicine faculties (with 07 faculties) whereas only 2 Surgical faculties have inadequate nurses. These understaffed faculties should be completed between 01 and 02 nurses each.

In this study, by selecting the same level hospitals (provincial level) and by selecting faculties in the Surgical and Internal Medicine block, we can use the results to compare the available and needed staff of faculties with the same specialization among hospitals by looking at the standard time for performing specific activities across the faculties within one hospital and across the selected hospitals. The findings showed that the AWT of hospitals ranges from 1850 to 2000 h per year, varies by hospital due to the difference in number of days off of faculties. The results showed that there was a substantial difference in the hours worked by nurses between hospitals and between the departments of the same hospital. The AWT of medical staff in Vietnam differs from other Africa countries. In some hospitals in Uganda, for example, the hours available for medical staff ranged between 1624 and 1688, or between 1825.4 and 1850 [12]. In fact, according to the number of the Labor Code 2012 (issued according to Decree 106/2012 dated 20/12/2012) [13], public servants, public service employees, work 8 h and 5 days per week, so every week has 40 h of work. However, in many faculties due to the great work pressure, staff often have to go half an hour earlier and go back home half an hour late to finish the job.

The proportion of time that nurses spend on clinical and administrative services varies between hospitals and faculties. The findings showed that administrative/management time represents 20–40% of a nurse's total work time. The outcome also revealed to the real context in hospitals that this is a waste or misuse of valuable nursing time. While nurses are trained in patient care, in fact, many are entrusted with activities other than care (e.g. paper working, medical records preparation, completion of transfer or discharge procedures in hospital). Our findings mirros the result from previous study in Nigeria [5], Indonesia [14] to indicate that the review of practice in health workforce should be implemented to ensure that the scope of practice of cadres match the specific training and skills and those are used in delivery of quality health services.

In this study we found that most faculties have two cadres of nursing staff, including caregiver (provide patient care) and administrative nurse (doing task of paying, and receiving and distributing drugs). The results show that some faculties are short of nursing staff, but, during this time, the work of administrative nurses is relatively light, with little or no pressure. As a result, time allocated to clinical services accounted for two-thirds of nurses' working time compared to time spent on administrative activities. As a result, the conclusion suggested that the review of the distribution of tasks in each faculty within the hospital should be implemented on a regular basis to ensure the efficient use of human resources. This study with WISN approach also helps managers to consider the consequences of human resource shortages, such as the quality of the medical service. When the WISN rate is high, it poses a difficult question to the manager to see if there is a staff shortage or if performance standards are problematic.

The recent Nigerian study also suggested examining scopes of practice to ensure that managers have the capacity to deliver quality service [5]. Furthermore, the standardization of professional and administrative activities, as well as the time needed to perform specific activities, should be reviewed. This would facilitate the proper distribution of tasks among managers that take into account skills, shortages and the inequitable distribution of the health workforce and current workload among the hospital faculties [15].

The WISN application obviously helps hospital managers to identify the lack or excess of human resources in the faculties, as well as to demonstrate executives with the strongest pressure; and maybe even come up with more suitable and effective human rearrangement solutions; and perhaps even find more appropriate and efficient human rearranging solutions. Based on these findings, the Can Tho central hospital, for example, provides an evidence base for decision-making in the reorganization and planning for the future recruitment of nurses to the hospital.

Our findings informed decision by the health policy makers to consider the current traditional staffing norms stated in the Circular 08/TT-BYT issued in 2007 [10] in which the staffing norms are applied to all hospitals of different levels (central-provincial-district) and merely based on the number of beds regardless other important factors such as population density, mortality and morbidity patterns with limited consideration to the characteristics of each faculty and hospital. The results from our study showed that the staffing need of faculties within one hospital and among hospitals are varied depending on the organization of work and task distribution in each faculty. The application of WISN therefore is recommended to scale up conduct of WISN study in more provinces across the country and encourage health managers and leaders to apply WISN as a national strategy for efficient health workforce planning.

### Limitation of the study

One of the limitations of this study is to simply predict and plan the need for clinical nurses in four selected hospitals. The study was carried out on the nursing framework in the departments of internal medicine and surgery of 4 provincial hospitals, so the generalization to other hospitals may be limited. These results should be applied with caution, as staff organization and assignment may vary from hospital to hospital. The application of WISN in different types of clinical services in a greater number of hospitals requires further study. The workload of nurses in charge of receiving and distributing drugs to patients and perform hospital discharge payments has not been accurately calculated, while in each faculty, there are an average of 3–4 nurses working in this position. The assessment of staff workload and the calculation of staff needs depend greatly on the availability and accuracy of existing data. For departments that have an adequate archiving or reporting system, this will be an obstacle to implementing WISN. Poor documentation of health services data at the institutional level has led to overestimation or underestimation of the staffing requirements of some health facilities.

## Conclusions

When applying WISN calculation, the study indicated that 10 among 22 faculties lacking from 1–2 nurses, the workload pressures in these faculties are not high with the WISN ratios are ranging from 0.88 to 0.95. Only 01 faculty of internal medicine from Can Tho Central Hospital lacks 05 nurses, facing a high workload with a WISN ratio of 0.78. It can be seen that the application of WISN has enabled health managers to analyze the workload of their cadres, calculate staffing needs, and thus effectively contribute to the workforce planning process. This study contributed to provide important evidence to help policy makers develop guidelines for personnel norms for health facilities in the context of limited resources, while the existing regulation Circular 08/TT-BYT since 2007 is no longer appropriate in Vietnam.

## Supplementary Information

**Additional file 1.** WISN dataset at four Vietnam hospitals.

## Data Availability

Data and materials are available on request.

## Abbreviations

AS: Activity Standard
AWT: Available Working Time
CAF: Category Allowance Factor
CAS: Category Allowance Standard
FGD: Focused Group Discussion
IAF: Individual Allowance Factor
IAS: Individual Allowance Standard
LMICs: Low and Middle Income Countries
WHO: World Health Organization
WISN: Workload Indicators of Staffing Need
